# Xiphodynia: A diagnostic conundrum

**DOI:** 10.1186/1746-1340-15-13

**Published:** 2007-09-15

**Authors:** J Keith Simpson, Erin Hawken

**Affiliations:** 1 Clinic Director, Murdoch University Chiropractic Clinic, School of Chiropractic, Murdoch University, Murdoch, Western Australia 6150, Australia; 2 Private Practice, Burswood Health Professionals, 21 Harvey Street, Burswood, Western Australia 6100, Australia

## Abstract

This paper presents 3 case reports of xiphodynia that presented to a chiropractic clinic. The paper examines aspects of xiphodynia including relevant anatomy of the xiphoid, as well as the incidence, aetiology, symptoms, diagnosis, and treatment. A brief overview of the mechanism of referred pain is presented.

## Background

Xiphodynia is a term used to describe an 'uncommon' syndrome with a constellation of symptoms ranging from upper abdominal pain, chest pain, sometimes throat and arm symptoms which are referred from the xiphisternal joint or the structures attached to the xiphoid process.

This paper offers 3 case reports of xiphodynia that presented to a chiropractic clinic. In the first two cases, during systems review it was revealed that the patients had ongoing 'organic' symptoms that had persisted for years and despite extensive investigations, no definitive diagnosis had been established and more importantly for the patients involved, no effective treatments administered. In the third case it was revealed that the patient could not lie prone and could not perform certain exercises because of the provocation of symptoms. The paper examines aspects of xiphodynia including relevant anatomy of the xiphoid, as well as the incidence, aetiology, symptoms, diagnosis, and treatment of xiphodynia. A brief overview of the mechanism of referred pain is also presented.

## Case I – mid-back pain following childbirth

DM, a 33 year old female presented to a chiropractic clinic with a chief complaint of neck pain and headaches following a motor vehicle accident and a secondary complaint of mid-dorsal pain. DM's primary complaint was diagnosed as Whiplash Associated Disorder (Grade II) (WAD II) and resolved following a course of spinal manipulative therapy (SMT), soft-tissue treatment (STT) and neck exercises. The onset of DM's second complaint of mid-dorsal pain was five years previously, following the difficult delivery of her first child. The pain had worsened following the birth of her second child, four years later. It was present every day, rated 7–8/10 at its worst and 3–4/10 at best. The mid-dorsal pain disturbed DM's sleep and physical activity aggravated the pain. Minor relief was obtained by application of moist heat over the painful thoracic area and stretching of the mid-back. Of note, DM had undergone a laparoscopic cholecystectomy between the pregnancies after which her mid-dorsal pain had completely abated for three weeks but then recurred. Aside from local tenderness in the mid-dorsal spine, physical examination was unremarkable. A course of manipulation and ultrasound to the mid-dorsal area was undertaken. Even though DM reported a feeling of increased mobility in her dorsal spine, the symptom of mid-back pain persisted. Because of the refractory nature of DM's mid-dorsal pain, re-assessment was performed. The reassessment included an abdominal examination because of the resolution of her symptoms following laparoscopic cholecystectomy. Examination revealed an exquisitely tender xiphoid process and reproduction of DM's mid-dorsal pain when pressure was applied to the left aspect of the xiphoid. A diagnosis of xiphodynia was established and a course of ultrasound (4 minutes, 2 watts/cm^2^, pulsed) undertaken over a two week period. Following five treatments DM's symptoms were significantly diminished but for unknown reasons, in the week between the fifth and sixth treatment, there was a relapse in mid-dorsal pain. At this point it was determined to refer her for an opinion from a Rheumatologist who performed a single injection of lidocaine and Celestone ™ to the spot found to be most tender to palpation in the xiphisternal area. DM's mid-dorsal pain resolved completely and has not returned in the intervening 9 years.

## Case II – abdominal pain and throat tightness following lifting

JS, a 25 year old female, presented to a chiropractic teaching clinic with a chief complaint of neck and upper back pain. Following the initial work-up, a diagnosis of neck pain of mechanical origin with an associated myofascial syndrome was made and a course of SMT, STT and exercises for the deep neck flexors undertaken. JS's symptoms were resolving well. During the course of a standard office visit it was noted that JS was being investigated for gastric ulcers because of a 3.5 year history of daily mild to severe and at times disabling abdominal pain with associated throat tightness. The pain would reach 8/10 in intensity and last for several hours. Repeated investigations including abdominal ultrasound, endoscopy, and tests for helicobacter pylori were all negative. JS's medical practitioner (GP) made a presumptive diagnosis of gastric ulcer disease and prescribed Cimetidine, then Lansoprazole and later Esomeprazole to no avail. JS was then advised by her GP to cease all medications and diagnosed functional dyspepsia. With persisting symptoms, the GP opted to prescribe an anti-depressant, Fluoxetine hydrochloride. JS's symptoms persisted and, in addition, she began to experience adverse effects to the Fluoxetine hydrochloride and so this was ceased. It was at this time that one of the authors (JKS) examined JS and discovered that both abdominal and throat symptoms were reproducible by direct pressure over the lateral aspect of her xiphoid process.

A diagnosis of xiphodynia was established and a course of treatment involving 2 minutes of low-level laser therapy (LLLT) to an area of approximately 4 cm^2 ^surrounding the xiphoid undertaken. A therapeutic trial of two laser treatments per week for up to four weeks was recommended. Progress was good. At the end of the third week JS reported significant decrease in frequency, duration and intensity of her abdominal pain. JS experienced days without any pain at all. In addition, the intensity of her pain was now reported as 3/10 maximum with duration of 30 minutes. Palpation of the xiphoid no longer reproduced abdominal pain although some throat tightness was still experienced. Two additional LLLT treatments were undertaken and JS was reviewed six weeks later at which time there was no tenderness over the xiphoid and JS reported infrequent very mild abdominal symptoms and no throat tightness. After reflecting upon her abdominal symptoms JS recalled that they began when she was working as a fruit-picker and had repeatedly performed awkward lifts of heavy crates. In order to lift and move the crates JS arched backward and used her abdomen to support the lift. This brought the edge of the crate into contact with her xiphoid process. The abdominal pain and throat tightness began the day following a particularly strenuous workday.

## Case III: abdominal pain, throat pain and headache

RH, a 21 year old female presented to a chiropractic teaching clinic with a chief complaint of neck and thoracic spinal pain which had been present for approximately 4 years since commencing her university studies and was aggravated by studying. RH experienced this pain about once per week and it lasted for 'a couple of days'. RH had a secondary complaint of headache, which began about the same time as her primary complaint. The headaches were described as a band of pain across her forehead along with an ache at the base of her skull and in her temples. Past medical history was unremarkable save for shingles in 2004. Routine physical examination was unremarkable. Following history and examination a working diagnosis of mid-back pain of mechanical origin with associated muscle hypertonicity was established and a course of chiropractic treatments proposed. Treatment included SMT to the spinal levels thought to have restricted motion, soft-tissue treatment to the musculature of the upper back and a recommendation for core stability exercises. On a subsequent visit RH reported that she could neither lie prone on the treatment table nor could she perform even the most basic core stability exercises because to do so would result in severe abdominal pain, throat tightness and headache. Following further questioning it was determined that RH had been experiencing this triad of symptoms since she was eight years old and had subsequently avoided any activity that placed pressure on her abdomen, such as sitting close to a table when studying or performing abdominal exercises. Medical investigation for her symptoms had yielded neither a diagnosis nor a treatment. RH traced her symptoms back to a ballet class when she was age eight years during which she was required to lie prone on a wooden floor and, while holding onto her ankle, attempt to touch her toes to her head. Palpation of the area surrounding RH's xiphoid reproduced her abdominal pain and throat tightness immediately with the headache beginning a few minutes later. These symptoms persisted for several hours. A diagnosis of xiphodynia was established and a course of LLLT suggested. Four treatments of 2 minutes each were administered. RH reported a lessening of her symptoms but not cessation. It was noted that, in order to reproduce the symptoms pressure had to be applied to the xiphoid for up to 20 seconds and that RH's symptoms would not begin for until two minutes after pressure was removed. A switch of modality to Ultrasound was commenced. Ultrasound was chosen because of its known effects on tissue repair [1,2]

In addition, RH was advised to use a topical anti-inflammatory gel over the xiphoid. Following four ultrasound treatments RH's symptoms persisted however they have subsided from 8/10 to 2–3/10 and she is content to continue with conservative care because she does not wish to receive an injection of corticosteroid to the area. RH was reviewed in April 2007 and related that she went overseas during the University summer break during which time her symptoms continued to subside. RH stated that her symptoms were mild and intermittent. At this point in time RH elected to have no examination or treatment of the xiphoid, instead opting to return for assessment and treatment if she experienced an exacerbation of symptoms.

## Discussion

### Xiphodynia

Xiphodynia is a condition involving referral of pain to the chest, abdomen, throat, arms and head from an irritated xiphoid process. The literature over a 60 year period reveals 12 citations relating to the terms xiphodynia and xiphoidalgia, with only 5 of these in English. The papers published between 1979 and 1998 [3-5] present 10 cases of xiphodynia, all treated by localized injection. Lipkin et al [6] published what appears to be the first 'modern' paper on the hypersensitive xiphoid in 1955. They reported on 24 cases observed over a seven year period where gentle pressure on a hypersensitive xiphoid reproduced all or most of the patients' presenting pain. Lipkin et al [6] note that the earliest report of disorders of the xiphoid was recorded in 1712.

### Incidence

There are no clear data relating to the incidence or prevalence of xiphodynia. Most authors say that it is an uncommon disorder [3-5,7] while Lipkin et al [6] found the syndrome present in about 2 percent of the population of a general-hospital ward and stated that it is "far more common than is generally appreciated". They went so far as to suggest that examination of the xiphoid should be part of the routine examination of any patient presenting with upper-abdominal or chest pain [6].

### Anatomy

**Figure 1:**
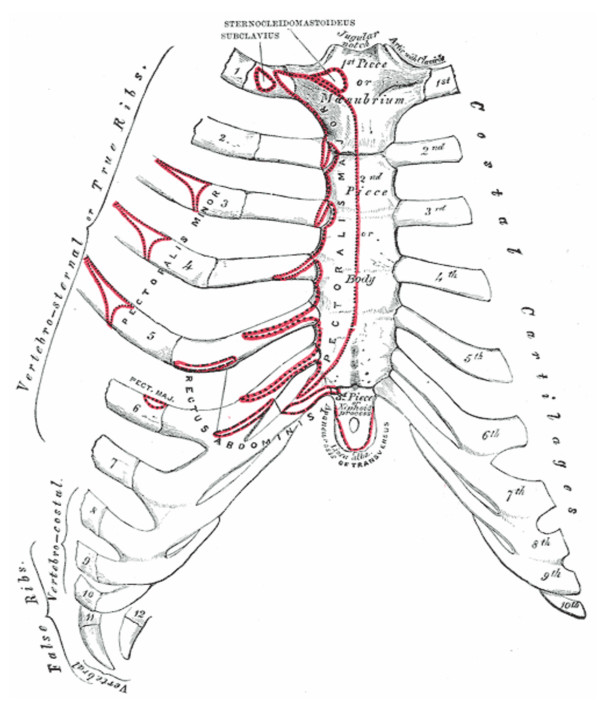
**The sternum – anterior surface [8]**. Figure 1 shows the anterior surface of sternum and costal cartilages. Muscular attachments are shown in red.

**Figure 2:**
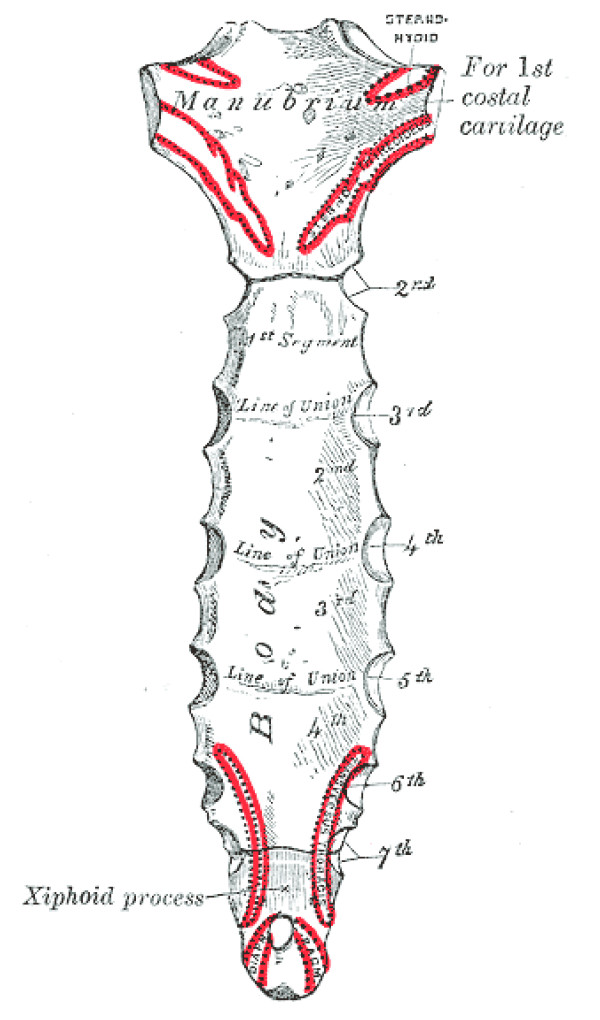
**The sternum – posterior surface [8]**. Figure 2 shows the posterior surface of the sternum. Muscular attachments are shown in red.

The xiphoid process is the smallest of the three sections of the sternum (see figures 1 and 2). It is a thin and elongated, cartilaginous in structure in youth, but becomes ossified at its upper part in the adult. The xiphoid may be broad and thin, pointed, bifid, perforated, curved, and may deviate laterally. The xiphoid forms a synchrondosis with the body of the sternum. On the front of each superior angle, there is a facet for part of the seventh costal cartilage.

The xiphoid process serves as an attachment for several soft tissue structures that have rich innervation. [8] (See Table 1)

**Table 1:** Sternal attachments and innervation [8]. Table 1 lists the soft-tissues that attach to the xiphoid and their innervation.

**Aspect**	**Attachment**	**Innervation**
Anterior	The Linea Alba Fibres of Rectus Abdominis	Lower intercostal nerves
Lateral	The aponeurosis of the three flat muscles (external oblique, internal oblique, and transversus abdominis)	Lower intercostal nerves, Obliquus internus and Transversus also receive filaments from the anterior branch of the iliohypogastric and sometimes from the ilioinguinal nerves
	Anterior costoxiphoid ligament	Lower intercostal nerves
Posterior	Posterior costoxiphoid ligament	Lower intercostal nerves
	Fibres from the diaphragm	Phrenic (C3,4,5), lower intercostal nerves
	Fibres from Transverse thoracis	Lower intercostal nerves

### Symptoms

Xiphodynia is a musculoskeletal disorder capable of producing a constellation of symptoms that mimic several common abdominal and thoracic diseases including:

○ Cardiac chest pain

○ Epigastric pain

○ Nausea, vomiting and diarrhoea

○ Radiating pain into the back, neck, shoulders, arms and chest wall [4]

### Aetiology

While xiphodynia is frequently insidious in onset, trauma may precipitate the syndrome. Acceleration/deceleration injuries [7], blunt trauma to the chest [7], unaccustomed heavy lifting and aerobics have been known to precipitate xiphodynia [4] likely because of the muscular attachments. The cases presented here all gave a history of 'trauma' which appeared to be associated with the onset of symptoms.

### Diagnosis

The diagnosis of xiphodynia is dependent upon the reproduction of the patient's symptoms completely or in part by moderate pressure on the xiphoid process and its adjacent structures.

Even though xiphodynia often exists in the absence of any other medical condition, it has been demonstrated in conjunction with life-threatening disease such as cardiac disease including angina pectoris, myocardial infarction, and pericarditis [3,5]. It is therefore imperative that any patient presenting to a primary health care provider with acute chest or abdominal pain be carefully investigated to establish a diagnosis and treatment plan. Where appropriate, emergency medical care must be rendered. In cases where a clear medical diagnosis cannot be established, a simple provocative test may uncover a symptomatic xiphoid process and establish the diagnosis of xiphodynia. In those patients who receive treatment for an established 'medical condition' in whom symptoms persist, consideration might be given to examining for xiphodynia.

### Treatment

The literature suggests that xiphodynia is a self-limiting disorder to be treated with reassurance [3] or with analgesics, topical heat and cold, and an elastic rib belt [7]. It is clear from these and other reported cases [3] that xiphodynia may not be self-limiting. The medical 'treatment of choice' is an injection of local anaesthetic and steroid [2-6]. Xiphoid injection, while often curative, is not without risk of complications including pleural or peritoneal perforation, pneumothorax, or infection [3,7].

Conservative physical therapies are worth a trial, however no evidence exists for their effectiveness with xiphodynia.

### Referred Pain

Given the extent of symptom referral that is the hallmark of xiphodynia, it is relevant to briefly consider the topic of referred pain here. Pain referred from a distant structure is a real phenomenon, one that sometimes presents a clinical conundrum for practitioners and, at times, leaves patients suffering untreated pain needlessly. This is not new. In 1893 Mackenzie [9] wrote about the phenomenon of viscero-somato pain referral. Kellgren [10] recognized the limitations inherent in defining the origin of back pain and, following a series of experiments in the late 1930s, mapped patterns of referred pain from deep structures such as deep fascia, periosteum, and ligaments. In the late 1940s Travell and Rinzler mapped referral patterns from pectoral muscles that mimicked the symptoms of angina pectoris and myocardial infarction [11]. More recently, Travell and Simons [12] identified referral patterns from myofascial trigger points throughout the body.

Whatever the source of the pain, and however well accepted is the phenomenon of referred pain, there is little agreement on the pathophysiology of referred pain. At one point a simple 'convergence' theory was proposed but is not supported by clinical and experimental observations [13]. Recent experiments by Kosek and Hansson [14] suggest that referred pain is "most likely a consequence of misinterpretation of the origin of input from the stimulated focal pain area, due to excitation of neurones somewhere along the neuroaxis with projected fields in the referred pain area". This 'central sensitization' theory of referred pain appears to be the one most supported by research in the area [15]. Interestingly, this is essentially what Mackenzie [9] proposed in 1893.

While the preponderance of patients presenting in the chiropractic setting have chief symptoms obviously related to the neuromusculoskeletal system, uncommonly such symptoms may appear to be organic in origin [16]. The adage "When you hear hoof beats, think horses, not zebras" exemplifies diagnostic thinking. Common things are common but unless we think also of uncommon things, we sometimes mis-diagnose and fail in our duty of care to our patients. Perhaps Harley S Smyth, Surgical Director of the Freeman Centre in Clinical and Molecular Endocrine Oncology, University of Toronto, was closer to the mark when he said, "When you hear hoof beats, think horses before zebras" [17]. From this we take: when a patient presents with what appears to be organic symptoms – investigate. But when an organic aetiology has been excluded and symptoms persist; consideration should be given to a musculoskeletal cause that might respond to conservative care.

## Conclusion

Xiphodynia is a musculoskeletal disorder that can be responsible for extremely distressing and disabling upper abdominal, chest and/or throat symptoms. It is simple to diagnose and simple to treat. Given the principal author's experience, which concurs with Lipkin et al [6], it may be that xiphodynia is under-considered. Lipkin et al [6] went so far as to suggest, "palpation of the xiphoid area be done in most general physical examinations and certainly in patients complaining of pain in the chest or upper abdomen". A short course of non-invasive treatment such as low-level laser or ultrasound may be worth considering before more aggressive injection treatment is utilized.

## Competing interests

The author(s) declare that they have no competing interests.

## Authors' contributions

Both authors collaborated on the rationale and design of the paper and liaised with the collection of references. EH drafted the preliminary paper involving the second case.

JKS expanded the paper and detailed the other cases. Both authors read and approved the final manuscript.
